# A population-based cohort study of the epidemiology of light-chain amyloidosis in Taiwan

**DOI:** 10.1038/s41598-022-18990-3

**Published:** 2022-09-21

**Authors:** Hsin-An Hou, Chao-Hsiun Tang, Choo Hua Goh, Shih-Pei Shen, Kuan-Chih Huang, Hong Qiu, Sarah Siggins, Lee Anne Rothwell, Yanfang Liu

**Affiliations:** 1 Division of Hematology, Department of Internal Medicine, National Taiwan University Hospital, Zhongzheng District, Taipei City, 100 Taiwan; 2 School of Health Care Administration, College of Management, Taipei Medical University, No.172-1 Keelung Road, Section 2, Taipei, 106 Taiwan; 3 Epidemiology, Office of the Chief Medical Officer, Johnson & Johnson, 2 Science Park Drive, Singapore, 118222 Singapore; 4 Epidemiology, Office of the Chief Medical Officer, Johnson & Johnson, No. 2, Min Sheng W. Rd., Taipei City, 104 Taiwan; 5 Epidemiology, Office of the Chief Medical Officer, Johnson & Johnson, 1125 Trenton-Harbourton Road, Titusville, NJ 08560 USA; 6 Janssen Medical Affairs Asia Pacific, 66 Waterloo Road, North Ryde, NSW 2113 Australia; 7 Global Real World Evidence, GDCS, GCSO, Janssen Research & Development LLC, Raritan, USA

**Keywords:** Haematological cancer, Epidemiology, Cancer epidemiology

## Abstract

The incidence rate of AL (light-chain) amyloidosis is not known in Asia. We conducted a retrospective cohort study using the Taiwan National Healthcare Insurance Research database and Death Registry to estimate incidence and all-cause case fatality rates, and characteristics of patients with AL amyloidosis in Taiwan. All patients with confirmed, newly diagnosed AL amyloidosis from 01-Jan-2016 until 31-Dec-2019 were enrolled and followed up until dis-enrolment, death or study end (31-Dec-2019). There were 841 patients with newly diagnosed AL amyloidosis with median age of 61.4 years and 58.7% were men. At diagnosis, cardiac, renal and liver-related diseases were present in 28.54%, 23.19% and 2.14% of patients, respectively. AL amyloidosis age-adjusted annual incidence was 5.73 per million population in 2016 and 5.26 per million population in 2019. All-cause case fatality ranged from 1.7 to 2.9% over the study period and was highest (~10%) in patients ≥ 80 years. Survival was significantly lower in patients with co-morbid cardiac, renal, or liver-related diseases which could indicate organ involvement. The incidence of AL amyloidosis in Taiwan appears to be similar to Western countries. The poor prognosis in patients with co-morbid diseases highlights the need for earlier diagnosis.

## Introduction

Light-chain (AL) amyloidosis is one of the most common presentations of amyloid disease and is caused by accumulation of misfolded monoclonal immunoglobulin light chains produced by plasma cells^1^. AL amyloidosis usually occurs in older adults and the symptoms are typically non-specific and of insidious onset. Consequently, the diagnosis of AL amyloidosis may be delayed^1^. Common presenting symptoms include dyspnea, weakness, orthopnea, pitting oedema, polyneuropathy and macroglossia^2^. The presence of cardiac, renal, or liver involvement at diagnosis is a strong predictor of early mortality in AL amyloidosis^3,4^. Multi-system involvement is common, and the majority of patients have cardiac or renal involvement at diagnosis. Cardiac pathology is a result of infiltration of light chains into the cardiac extracellular space, causing biventricular wall thickening, ventricular remodeling, and restrictive cardiomyopathy^5^. Infiltration of the kidneys by amyloid results in proteinuria which can progress to nephrotic syndrome and end-stage kidney disease^6^. Survival is variable and depends primarily on the level of cardiac dysfunction, ranging from several months in patients who present with extensive cardiac involvement, to more than 10 years in patients with early-stage disease and favorable prognostic indicators^7,8^. The prognosis is also influenced by the bone marrow plasma cell burden, level of immunoparesis, and the presence of specific genetic markers that are predictive of a response to therapy^6^.

Although one of the most common forms of amyloid disease, AL amyloidosis is rare. The annual incidence is thought to be around 10 per million population and appears to be unchanging over time^9,10^. Population-based clinical information about AL amyloidosis incidence and mortality is limited^10,11^, and data in Asia are particularly lacking. Treatment options for AL amyloidosis are expanding as proteasome inhibitors, immunomodulators, monoclonal antibodies and small molecule inhibitors enter clinical trials^12^. Reference population-based information about the disease burden will be needed to support health technology assessments evaluating the impact of new treatments. We conducted a retrospective population-based cohort study to describe the clinical characteristics and epidemiology of patients with AL amyloidosis in a real-world setting in Taiwan. The study objectives were to estimate the annual incidence and fatality of newly diagnosed AL amyloidosis by age and gender, and to document the demographic and clinical characteristics of patients with newly diagnosed AL amyloidosis during the study period.

## Methods

### Data source

Patients with AL amyloidosis were identified from the National Health Insurance Research Database (NHIRD) in Taiwan. The NHIRD is a population-based claims database that holds information on all medical services provided throughout Taiwan. Diagnoses have been coded using the International Classification of Diseases, Tenth Revision (ICD-10) from 01 January 2016. The database holds information on patient demography, the date and type of service provided including physician services, diagnoses, prescriptions, inpatient and outpatient episodes, and claims information of laboratory and imaging examinations. Deaths were identified from the Death Registry which is linked to the NHIRD at the patient level using scrambled identification codes.

De-identified, aggregated patient data were used for the analysis. The study was granted an exemption from ethical review by the Taipei Medical University-Joint Institutional Review Board, and an exemption from the need for patient consent. The study was conducted according to all applicable guidelines and regulations set by the Health and Welfare Data Science Center (HWDC).

### Study population

All patients with newly diagnosed AL amyloidosis in the NHIRD (ICD-10 E85.4: Organ-limited amyloidosis, E85.8: Other amyloidosis and E85.9: Amyloidosis, unspecified) were enrolled from 1 January 2016 to 31 December 2019. Eligible patients had a biopsy record within 12 months prior to, or up to 6 months after the date of diagnosis of AL amyloidosis; and at least two consecutive outpatient claims and/or one inpatient claim for AL amyloidosis in the NHIRD. The biopsy codes included were specific to organs and sites that AL amyloidosis would affect, such as bone marrow, heart, gastrointestinal organs, kidney and skin.

Patients were excluded if the initial diagnosis switched to another amyloidosis code (ICD 10: E85.0, E85.1, E85.2, E85.3, E83.3) during the follow-up period. Patients were also excluded if they had a record of non-AL amyloidosis (ICD9: 277.3 Familial Amyloidotic Polyneuropathy; ICD-10: E85.1 neuropathic heredofamilial amyloidosis) in the Catastrophic Illness database. Registration in the Catastrophic Illness database is contingent upon meeting strict diagnostic evaluation criteria including biopsy confirmation and evaluation by two experts, and patients included in the database can be considered as having a confirmed diagnosis.

### Outcomes

The index date was the date of the first diagnosis of AL amyloidosis or the date of the first biopsy, whichever occurred first. Patients were followed-up until death, dis-enrolment, or the 31 December 2019, whichever occurred first.

Patient demographic characteristics, index year of diagnosis and Charlson-Co-morbidity Index (CCI) score were captured and assessed. Cardiac, liver and renal-related co-morbidities were identified using ICD-10 codes and were confirmed by having at least one inpatient hospitalization claim and/or at least two outpatient claims (within 12 months and with intervals of > 1 month between two claims).

### Statistical analysis

Annual incidence and all-cause case fatality rates were calculated by age and sex with 95% confidence intervals (CI). Age standardization used the World Health Organization world population 2000–2025. The all-cause case fatality rate was calculated by dividing the number of deaths in patients with AL amyloidosis from any cause by the total number of patients with AL amyloidosis in each calendar year. A Cox proportional hazard model was used to estimate hazard ratios (HR) of all cause death in patients with and without co-morbid cardiac, renal, and liver-related diseases. Potential confounders that could affect patient survival, such as age and sex. Survival was estimated for patients with and without cardiac, liver, and renal co-morbidities using regression-adjusted survival curves. The same confounders were adjusted to describe case fatality in patients with different co-morbidities. Chi-square tests were used to compare frequency distributions between groups. A p value <0.05 was considered statistically significant. All analyses were performed using SAS Version 9.4 (Cary, NC, USA).

## Results

### Demographic and disease characteristics

**Figure 1:**
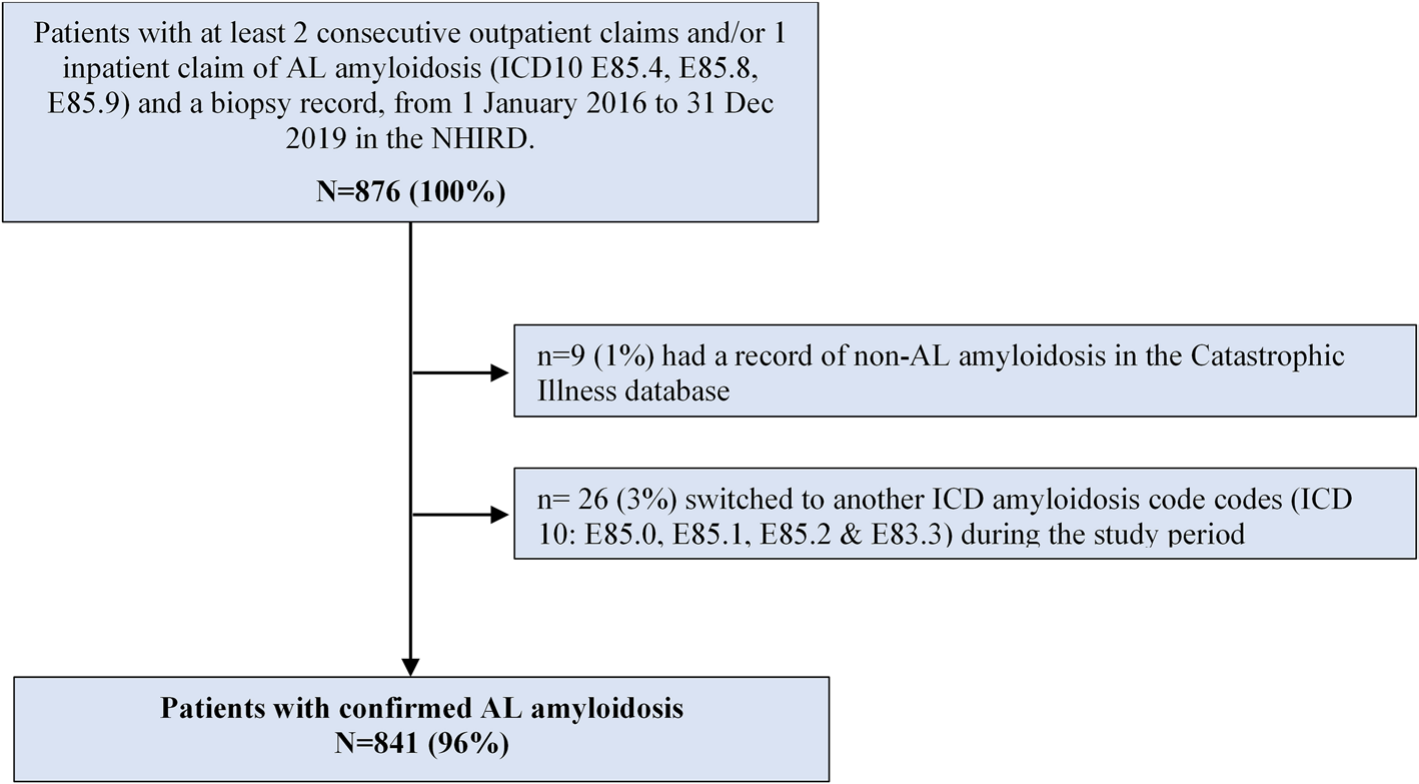
Patient enrollment flowchart. CD10: International Classification of Diseases 10th version NHIRD, National Health Insurance Research Database.

There were 876 patients who met the study inclusion criteria, of whom 9 had a record of non-AL amyloidosis in the Catastrophic Illness database, and 26 switched to another ICD amyloidosis code during the study period (Fig. 1). After applying exclusion criteria there were 841 patients with newly diagnosed AL amyloidosis between 2016 and 2019, of whom 58.74% (494/841) were men (Table S1). The mean age was 61.4 years (standard deviation [SD] 14.09, range 11 to 94 years), and 81.1% of patients were aged 50 years or older at the time of diagnosis. More than half of patients (51.6%) had CCI scores of ≥ 2. The most common co-morbidities were malignancy (21.9%), peptic ulcer disease (19.5%), renal disease (19.4%), and diabetes without chronic complications (19.1%) (Table S2). Cardiac-related co-morbidities were present in 28.54% of patients, of whom 20.69% had cardiac failure, 10.58% had a conduction disorder and 4.76% had cardiomyopathy. Renal-related co-morbidities were present in 23.19% of patients and included chronic kidney disease (14.15%). Liver-related diseases were present in 2.14% of patients (Table 1). Among 240 patients with cardiac-co-morbidities, 15 (6.25%) were between the ages of 20–49 years and 51 (21.25%) were 50–59 year of age. 73% patients with cardiac disease were aged 60 years and over (Table S2). Demographic and disease features were similar in patients enrolled across each calendar year (Table S1).

**Table 1:** Demographic and disease characteristics of patients with an AL amyloidosis claim in the NHRID who did, and did not, undergo biopsy during 2016–2019 in Taiwan.

	With biopsy (N = 841)		Without biopsy (N = 4442)		*p*
	N	%	N	%	
**Sex**					0.1338
Male	494	58.74	2466	55.52	
Female	347	41.26	1937	43.61	
missing			39	0.88	
**Age, years**					
Mean ± SD	61.40	14.09	56.37	16.82	< 0.0001
0–9	0		10	0.23	< 0.0001
10–19			51	1.15	
20–29	23 (10–29)	2.74	229	5.16	
30–39	38	4.52	526	11.84	
40–49	98	11.65	723	16.28	
50–59	219	26.04	980	22.06	
60–79	227	26.99	977	21.99	
70–79	159	18.91	581	13.08	
80 +	77	9.16	365	8.22	
**CCI score**					
Mean ± SD	2.07	2.23	0.82	1.37	< 0.0001
0	262	31.15	2689	60.54	< 0.0001
1	145	17.24	826	18.60	
2	162	19.26	431	9.70	
3	98	11.65	253	5.70	
4 +	174	20.69	243	5.47	
**Co-morbidities**					
Cardiac-related conditions	240	28.54	546	12.29	< 0.0001
Heart failure	174	20.69	393	8.85	< 0.0001
Conduction disorder	89	10.58	165	3.71	< 0.0001
Myocardial infarction	11	1.31	25	0.56	0.0160
Cardiomyopathy	40	4.76	7	0.16	< 0.0001
Liver-related diseases	18	2.14	10	0.23	< 0.0001
Renal-related diseases	195	23.19	262	5.90	< 0.0001
Chronic kidney disease	119	14.15	185	4.16	< 0.0001
End-stage renal disease	58	6.90	96	2.16	< 0.0001
Pleural effusion	55	6.54	8	0.18	< 0.0001
Neuropathy	22	2.62	46	1.04	0.0002
Malignancy*	192	22.83	177	3.98	< 0.0001
Solid tumor malignancy	113	13.44	157	3.53	< 0.0001
Hematological malignancy	99	11.77	35	0.79	< 0.0001
Multiple myeloma	66	7.85	11	0.25	< 0.0001
**Died**	166	19.74	228	5.13	< 0.0001

*CCI* Charlson Co-morbidity Index, *SD* standard deviation,

*Any malignancy including lymphoma and leukemia, except malignant neoplasm of skin.

There is no specific code for AL amyloidosis either in ICD-9 or ICD-10. To assess the robustness of our diagnostic criteria we evaluated the proportion of patients included in the study who had received at least one treatment for AL amyloidosis (chemotherapy, proteasome inhibitor, immunomodulatory agent, radiation therapy, steroids, autologous stem cell transplantation) during the follow-up period. There were 66.6% of patients who had received at least one relevant treatment. However, no treatment may be indicated in patients with mild disease^13^, and novel agents such as bortezomib were not reimbursed by the Taiwan NHI at the time of the study, and thus were not captured in the NHIRD if they were prescribed. Therefore, treatment history could not to be used to confirm the AL amyloidosis diagnosis in our cohort.

We secondly assessed the robustness of our diagnostic criteria by evaluating the demographic characteristics, disease features and case fatality among the population of 4442 patients who did not undergo confirmatory biopsy after receiving an initial diagnosis of AL amyloidosis (Table 1). Compared with patients who had a biopsy, patients with an AL amyloidosis diagnosis who did not undergo biopsy were significantly younger (mean 61.4 years vs. 56.4, *p* < 0.0001), had significantly fewer co-morbidities (mean CCI scores 0.82 vs. 2.07, *p* < 0.0001), had significantly lower incidences of all co-morbidities, and a significantly lower proportion died during the follow-up period (5.13% vs. 19.74%, *p* < 0.0001). These data suggest that patients with an initial diagnosis of AL amyloidosis who did not undergo confirmatory biopsy later received an alternative diagnosis. This is not unexpected given the non-specific clinical presentation of amyloid disease which can mimic many other conditions. The results provided some confidence that the inclusion criteria of ICD code plus evidence of a biopsy increased the specificity of the AL amyloidosis diagnosis in the database. However, we cannot exclude that a fraction of cases without confirmatory biopsy were patients with AL amyloidosis patients who did not undergo a biopsy for whatever reason.

### Incidence and case fatality rates

**Figure 2:**
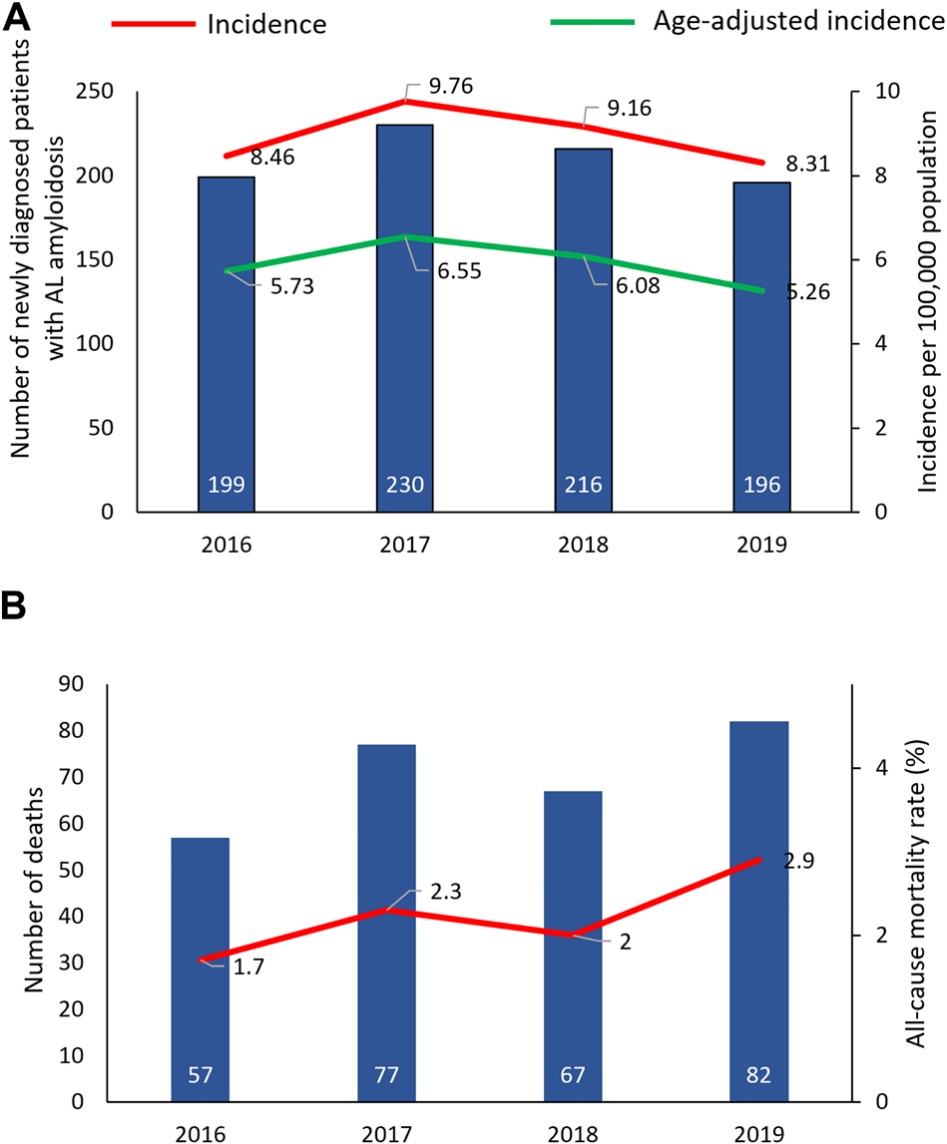
Crude and age-standardized (**A**) incidence and (**B**) all-cause case fatality rates in patients with AL amyloidosis in Taiwan 2016–2019.

The age-adjusted annual incidence of AL amyloidosis was 5.73 per million population in 2016, 6.55 per million population in 2017, 6.08per million population in 2018 and 5.26 per million population in 2019 (Fig. 2A). All-cause case fatality fluctuated from 1.7% in 2016 to 2.9% in 2019 (Fig. 2B).

### Impact of age and sex on incidence and all-cause case fatality rate

The incidence rates of AL amyloidosis increased with age and were similar in men and women until the age of 50 years, after which incidence rates increased more markedly in men than women (Table 2). While the incidence rate increased little in women after the age of 60 years, it continued to increase in men until ≥ 70 years of age. Over the observation period, the incidence rates of AL amyloidosis ranged from 9.65 to 11.78 per million population in men (all ages) and from 6.36 to 8.18 per million population in women (all ages).

**Table 2:** Sex-specific and age-specific incidence rate (with 95% confidence interval) of AL amyloidosis in Taiwan (age-standardized rates per 1,000,000 population) 2016–2019.

Age (yrs)	2016			2017			2018			2019		
	Total	Male	Female	Total	Male	Female	Total	Male	Female	Total	Male	Female
Total	8.46 (7.33–9.72)	10.58 (8.80–12.62)	6.36 (5.00–7.97)	9.76 (8.54–11.11)	11.78 (9.89–13.91)	7.77 (6.27–9.53)	9.16 (7.98–10.47)	10.16 (8.41–12.15)	8.18 (6.63–9.97)	8.31 (7.18–9.55)	9.65 (7.95–11.60)	6.98 (5.56–8.66)
< 20	0.00 (0.00–0.80)	0.00 (0.00–1.53)	0.00 (0.00–1.66)	0.22 (0.00–1.23)	0.00 (0.00–1.57)	0.46 (0.01–2.58)	0.00 (0.00–0.84)	0.00 (0.00–1.61)	0.00 (0.00–1.75)	0.00 (0.00–0.85)	0.00 (0.00–1.64)	0.00 (0.00–1.78)
20–29	2.19 (0.88–4.51)	2.41 (0.66–6.18)	1.95 (0.40–5.68)	2.80 (1.28–5.31)	2.40 (0.65–6.14)	3.23 (1.05–7.54)	0.63 (0.08–2.27)	0.60 (0.02–3.37)	0.65 (0.02–3.63)	1.28 (0.35–3.26)	0.61 (0.02–3.42)	1.99 (0.41–5.81)
30–39	1.55 (0.57–3.38)	2.08 (0.57–5.32)	1.03 (0.12–3.72)	2.65 (1.27–4.88)	2.66 (0.86–6.21)	2.64 (0.86–6.16)	3.51 (1.87–6.01)	3.25 (1.19–7.07)	3.78 (1.52–7.79)	2.49 (1.14–4.73)	2.76 (0.90–6.45)	2.22 (0.60–5.68)
40–49	7.15 (4.67–10.47)	10.57 (6.36–16.50)	3.81 (1.53–7.84)	5.73 (3.54–8.75)	3.87 (1.56–7.97)	7.53 (4.12–12.64)	8.93 (6.15–12.54)	7.15 (3.81–12.22)	10.66 (6.51–16.47)	4.83 (2.86–7.63)	5.45 (2.62–10.03)	4.22 (1.82–8.32)
50–59	13.54 (10.02–17.90)	18.52 (12.75–26.01)	8.71 (4.98–14.15)	15.70 (11.89–20.34)	19.03 (13.18–26.59)	12.47 (7.90–18.71)	18.14 (14.03–23.08)	20.68 (14.56–28.50)	15.68 (10.50–22.52)	12.90 (9.48–17.16)	15.65 (10.40–22.62)	10.25 (6.17–16.01)
60–69	23.22 (17.72–29.88)	24.90 (16.92–35.35)	21.65 (14.50–31.09)	23.74 (18.32–30.26)	33.41 (24.28–44.85)	14.78 (9.15–22.59)	18.14 (13.55–23.79)	19.61 (12.93–28.54)	16.78 (10.86–24.78)	16.76 (12.44–22.10)	21.66 (14.72–30.75)	12.24 (7.37–19.12)
70–79	25.01 (16.99–35.50)	33.78 (20.34–52.74)	17.73 (9.16–30.97)	34.09 (24.67–45.92)	48.88 (32.48–70.64)	21.79 (12.19–35.93)	29.08 (20.58–39.91)	47.07 (31.28–68.03)	14.04 (6.73–25.83)	34.25 (25.16–45.54)	46.31 (31.02–66.51)	24.12 (14.30–38.12)
≧80	27.20 (16.61–42.01)	41.92 (22.92–70.33)	14.95 (5.49–32.54)	31.77 (20.35–47.26)	47.61 (27.21–77.31)	19.07 (8.23–37.58)	15.42 (7.97–26.93)	20.61 (8.29–42.47)	11.39 (3.70–26.59)	26.12 (16.17–39.93)	26.13 (11.95–49.59)	26.12 (13.50–45.63)

The all-cause case fatality rate was similar in men and women and increased similarly from age 40 years in men and women (Table 3), The highest all-cause case fatality rates, around 10%, were observed in patients aged 80 years and older.

**Table 3:** Sex-specific and age-specific all-cause case fatality rate (with 95% confidence interval) of AL amyloidosis in Taiwan (age-standardized rates per 100 patients with AL amyloidosis) 2016–2019.

Age (yrs)	2016			2017			2018			2019		
	Total	Male	Female	Total	Male	Female	Total	Male	Female	Total	Male	Female
Total	1.73 (1.31–2.24)	2.09 (1.49–2.85)	1.26 (0.75–1.99)	2.29 (1.81–2.86)	2.83 (2.14–3.65)	1.53 (0.95–2.33)	2.00 (1.55–2.53)	2.06 (1.48–2.79)	1.90 (1.24–2.77)	2.87 (2.29–3.55)	3.13 (2.35–4.09)	2.51 (1.70–3.56)
< 20	0.00 (0.00–18.53)	0.00 (0.00–33.63)	0.00 (0.00–33.63)	0.00 (0.00–16.84)	0.00 (0.00–40.96)	0.00 (0.00–24.71)	0.00 (0.00–15.44)	0.00 (0.00–28.49)	0.00 (0.00–28.49)	0.00 (0.00–24.71)	0.00 (0.00–33.63)	0.00 (0.00–60.24)
20–29	0.00 (0.00–3.39)	0.00 (0.00–7.25)	0.00 (0.00–6.16)	0.88 (0.02–4.79)	1.67 (0.04–8.94)	0.00 (0.00–6.60)	0.00 (0.00–3.36)	0.00 (0.00–5.52)	0.00 (0.00–8.22)	0.00 (0.00–3.33)	0.00 (0.00–6.85)	0.00 (0.00–6.27)
30–39	0.00 (0.00–1.03)	0.00 (0.00–2.07)	0.00 (0.00–2.03)	0.00 (0.00–1.09)	0.00 (0.00–1.93)	0.00 (0.00–2.48)	0.00 (0.00–1.15)	0.00 (0.00–2.04)	0.00 (0.00–2.60)	0.00 (0.00–1.54)	0.00 (0.00–2.86)	0.00 (0.00–3.27)
40–49	0.18 (0.00–1.00)	0.41 (0.01–2.25)	0.00 (0.00–1.19)	0.53 (0.11–1.54)	1.10 (0.23–3.19)	0.00 (0.00–1.24)	0.64 (0.17–1.63)	0.95 (0.20–2.76)	0.32 (0.01–1.77)	0.21 (0.01–1.14)	0.00 (0.00–1.49)	0.42 (0.01–2.30)
50–59	1.08 (0.47–2.11)	1.62 (0.60–3.49)	0.54 (0.07–1.93)	0.93 (0.38–1.91)	1.04 (0.28–2.64)	0.82 (0.17–2.38)	1.32 (0.60–2.48)	1.88 (0.76–3.84)	0.64 (0.08–2.30)	1.58 (0.72–2.98)	2.08 (0.77–4.48)	1.06 (0.22–3.08)
60–69	1.61 (0.83–2.80)	1.71 (0.74–3.33)	1.45 (0.40–3.67)	2.09 (1.20–3.37)	2.41 (1.25–4.18)	1.49 (0.41–3.78)	2.00 (1.19–3.15)	1.79 (0.86–3.27)	2.35 (1.02–4.58)	2.20 (1.28–3.49)	2.63 (1.41–4.45)	1.43 (0.39–3.63)
70–79	3.16 (1.74–5.25)	2.74 (1.19–5.33)	3.97 (1.47–8.45)	3.62 (2.12–5.73)	3.76 (1.96–6.48)	3.31 (1.08–7.56)	5.04 (3.10–7.67)	4.68 (2.51–7.86)	5.88 (2.40–11.74)	7.89 (5.39–11.08)	7.48 (4.56–11.44)	8.73 (4.44–15.08)
≧80	6.75 (4.28–10.04)	6.32 (3.66–10.07)	8.22 (3.08–17.04)	9.94 (6.94–13.68)	9.49 (6.17–13.79)	11.39 (5.34–20.53)	5.30 (3.06–8.46)	3.81 (1.66–7.37)	8.70 (3.83–16.42)	8.74 (5.74–12.63)	7.41 (4.11–12.12)	11.34 (5.80–19.39)

In the Cox proportion regression model, the risk of all-cause death was higher in men than in women (HR 1.49 [95% CI 1.07–2.09]; *p* = 0.019) and increased with age. The risk of death was 6.9-fold higher in patients > 70 years of age with AL amyloidosis than 18 to 49 year-olds with AL amyloidosis (HR 6.9 [95% CI 2.98–15.92]; *p* < 0.0001) (Table 4).

**Table 4:** Adjusted cox proportion regression model for all-cause deaths among patients with AL amyloidosis in Taiwan.

	Parameters estimate	Hazard ratio	95% CI	*p*
**Gender**				
Female	Ref			
Male	0.4002	1.492	1.068–2.085	0.0191
**Age group**				
18–49 years	Ref			
50–59 years	1.0942	2.987	1.238–7.206	0.0149
60–79 years	1.5817	4.863	2.076–11.392	0.0003
> 70 years	1.9288	6.881	2.975–15.916	< 0.0001

*CI* confidence interval.

### Impact of cardiac, renal and liver-related co-morbidities on all-cause deaths

We evaluated the risk of all-cause death in patients with only cardiac and/or renal co-morbidities or with liver co-morbidities against a reference group without any co-morbidities at diagnosis. The risk of death was higher in patients with co-morbid disease and the highest risk of all-cause death was associated with co-morbid liver disease (HR 13.509, 95% CI, 7.491–24.363; *p* < 0.0001), followed by renal disease (HR 2.846, 95% CI 1.815–4.462; *p* < 0.0001), and cardiac disease (HR 2.007, 95% CI 1.299–3.102; *p* = 0.0017). Survival was lower again among patients with co-morbid cardiac and renal disease compared to patients with single organ (cardiac or renal) disease (HR 4.009, 95% CI 2.541–6.325; *p* < 0.0001).

**Figure 3:**
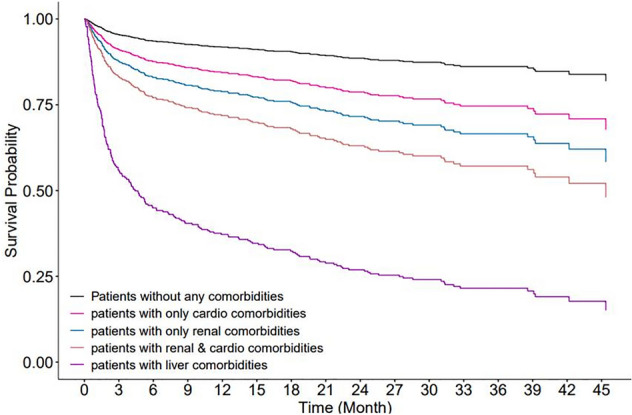
Regression-adjusted survival curves for all-cause death in patients with AL amyloidosis in Taiwan adjusted for age and sex. Among 149 patients with only cardiac disease, 105 (12.5%) had heart failure, 55 (6.5%) had a conduction disorder, 26 (3.06%) had cardiomyopathy, and 6 (0.7%) had myocardial infarction. Among 106 patients with only renal disease, 61 (7.25%) had chronic kidney disease and 31 (3.69%) had end-stage renal disease.

Six months after diagnosis, fewer than 50% of patients with liver-related co-morbidity had survived, whereas patients with cardiac and/or renal co-morbidity had not reached that threshold after 45 months of follow-up (Fig. 3).

## Discussion

To our knowledge, this is the first population-based cohort study to describe the epidemiology of AL Amyloidosis in Asia. Our study showed that AL amyloidosis is a rare disease in Taiwan, with age-adjusted incidence rates of 5.26 to 6.55 per million population. Incidence rates increased with age and tended to be similar in males and females until the age of 50 years, steadily increasing with age thereafter in males, but plateauing in females. There was no apparent change in the incidence of AL amyloidosis over the 4 years of the study.

Due to the rarity of the disease there have been few studies that report the epidemiology of AL amyloidosis. The incidence rate of AL amyloidosis was 5 per million population (age adjusted) when extrapolated from patients referred to the National Amyloidosis Centre in England (2008)^14^; 6.1 (95% CI 2.6–9.7) per million population (age and sex adjusted) from a patient registry in Argentina (2006 to 2015)^15^; 12.5 (95% CI 5.6–19.4) per million population (crude rate) in a population-based study in the Limousin region of France (2012 to 2016)^11^; 12 (95% CI 8–16) per million population (age and sex adjusted) in a population-based study in the United States (US) (1990 to 2015)^10^; and 10.8 to 15.2 per million population (age and sex adjusted) in the US using a healthcare claims database (2007 to 2015)^16^. Until now the epidemiology of AL amyloidosis in Asia has not been described. Our population-based study suggests that the incidence of AL amyloidosis in Taiwan is similar to western countries.

The demographic characteristics of patients with newly diagnosed AL amyloidosis were consistent with studies in other countries. We observed incidence rates that were 1.2 to 1.7-fold higher in males than in females, which compares favorably with a 1.6-fold higher incidence in men than women in the US^16^. The mean age at diagnosis was 61.4 years (median 62.2), which is lower than 72.5 years (median) reported in France^11^, 76 years (median) in the US between 1990 and 2015^10^, but closer to 64 years in the US between 2008 and 2015^16^, possibly suggesting that diagnosis has occurred earlier in recent years. Diagnostic delay is recognized as an important limitation in the treatment of AL amyloidosis but has been poorly quantified.

Older age had a strong association with an increased risk of death, independent of the number of co-morbid conditions. However, survival probability was significantly lower in patients with co-morbid cardiac, renal or liver disease. In our study, we used ICD-10 codes to identify patients with relevant co-morbid conditions as possible indicators of amyloid involvement of these organs. We recognize the limitations of this approach given that no clinical or biopsy data were available to confirm the underlying cause. In our study, cardiac disease was present at diagnosis in 28% of patients, renal disease in 23%, and liver disease in 2%. Cardiac involvement has been reported in 21% to 70% of patients with AL amyloidosis, renal disease in 48% to 100%, and liver disease in 15–20% of patients^2,4,17^, with evidence of some variation between Western and Eastern countries, although more data are needed to quantify potential differences^18^. The lower occurrence of cardiac, renal and particularly liver-related diseases in our population could be due to early diagnosis, ethnic differences in disease progression, or could reflect the lack of sensitivity of ICD codes where symptoms such as hypoalbuminemia or raised transaminases might not be specifically coded. Nevertheless, we found that the presence of cardiac, renal or liver disease was significantly associated with an increased risk of all-cause death in patients with AL amyloidosis. Co-morbid liver disease had most impact on prognosis, which is consistent with a study from China in which the median survival in patients with liver involvement was 9.2 months, and shorter than patients with either cardiac (19.3 months) or renal insufficiency (11.7 months)^17^.

Our study is the first real-world, population-based assessment of the epidemiology of AL amyloidosis in an Asian country. Strengths of the study are the completeness of data capture in the NHIRD and death registry, robust inclusion and exclusion criteria including biopsy confirmation of disease that maximized the accuracy of the diagnosis, and the recency of the data for a disease for which treatment options are rapidly evolving and impacting patient survival. Although mis-classification cannot be completely ruled out, the probability of misclassification was further reduced by selecting the cases with a biopsy-confirmed diagnosis of AL amyloidosis.

Potential limitations include the lack of clinical information in the claims database, and the relatively short study duration of 4 years, which was determined by the use of ICD-10 coding in the NHIRD and did not allow us to observe temporal trends in disease incidence or survival. In the absence of a specific disease code, identification of patients with AL amyloidosis in the database was challenging. We required several claims and a recent biopsy as evidence of AL amyloidosis and excluded patients who had a confirmed non-AL amyloidosis diagnosis in the Catastrophic Illness Registry. We were unable to use treatment codes to confirm the diagnosis but assessed the robustness of our definition by evaluating the characteristics of patients who met the study inclusion criteria by comparing against patients who did not have a biopsy performed. The demographic and clinical characteristics of this cohort were quite different from the study cohort, suggesting that a biopsy record improved the specificity of the AL amyloidosis diagnosis.

These data present the most recent picture of the epidemiology of AL amyloidosis and our incidence estimate is consistent with the recent estimates from Western countries. The lower frequencies of cardiac, renal, and liver co-morbidities in our study could be related to better survival noted in this cohort. However, the NHIRD database does not provide clinical information, and not all organ involvement may have been accurately captured.

In conclusion, the incidence of AL amyloidosis in Taiwan appears to be similar to Western countries. Potential differences in organ involvement between East and West could influence treatment choice and response to therapy and need further exploration. The poor prognosis associated with co-morbid cardiac, renal and liver diseases highlights the need for a high level of clinical suspicion to promote early diagnosis of AL amyloidosis, prior to infiltration of major organ systems.

## Supplementary Information

Supplementary Information.

## Data Availability

The data underlying this study are from the NHIRD which has been transferred to the HWDC. The Taiwan government prohibits release of the NHI claims dataset to the public domain. Interested researchers can obtain the data through formal application to the HWDC, Department of Statistics, Ministry of Health and Welfare, Taiwan (https://dep.mohw.gov.tw/DOS/cp-5119-59201-113.html).

## References

[1] Milani P, Merlini G, Palladini G (2018). Light chain amyloidosis. Mediterr. J. Hematol. Infect. Dis..

[2] Hwa YL (2019). Immunoglobulin light-chain amyloidosis: Clinical presentations and diagnostic approach. J. Adv. Pract. Oncol..

[3] Wechalekar AD (2013). A European collaborative study of treatment outcomes in 346 patients with cardiac stage III AL amyloidosis. Blood.

[4] Rosenzweig M, Kastritis E (2020). Liver and gastrointestinal involvement: Update. Hematol. Oncol. Clin. North Am..

[5] Martinez-Naharro A, Hawkins PN, Fontana M (2018). Cardiac amyloidosis. Clin. Med. (Lond).

[6] Dittrich T, Kimmich C, Hegenbart U, Schonland SO (2020). Prognosis and staging of AL amyloidosis. Acta Haematol..

[7] Palladini G, Milani P, Merlini G (2020). Management of AL amyloidosis in 2020. Hematology Am. Soc. Hematol. Educ. Prog..

[8] Muchtar E (2019). Ten-year survivors in AL amyloidosis: Characteristics and treatment pattern. Br. J. Haematol..

[9] Merlini G, Palladini G (2013). Light chain amyloidosis: The heart of the problem. Haematologica.

[10] Kyle RA (2019). Incidence of AL Amyloidosis in Olmsted County, Minnesota, 1990 through 2015. Mayo Clin. Proc..

[11] Duhamel S (2017). Incidence and prevalence of light chain amyloidosis: A population-based study. Blood.

[12] Zhang KW, Stockerl-Goldstein KE, Lenihan DJ (2019). Emerging therapeutics for the treatment of light chain and transthyretin amyloidosis. JACC Basic Transl. Sci..

[13] Mateos MV, San Miguel JF (2013). New approaches to smoldering myeloma. Curr. Hematol. Malig. Rep..

[14] Pinney JH (2013). Systemic amyloidosis in England: An epidemiological study. Br. J. Haematol..

[15] Aguirre MA (2016). Incidence rate of amyloidosis in patients from a medical care program in Buenos Aires, Argentina: A prospective cohort. Amyloid.

[16] Quock TP, Yan T, Chang E, Guthrie S, Broder MS (2018). Epidemiology of AL amyloidosis: A real-world study using US claims data. Blood Adv..

[17] Huang X (2015). The clinical features and outcomes of systemic AL amyloidosis: A cohort of 231 Chinese patients. Clin. Kidney J..

[18] Huang XH, Liu ZH (2016). The clinical presentation and management of systemic light-chain amyloidosis in China. Kidney Dis. (Basel).

