# Supplementary material for: A population-based cohort study of the epidemiology of light-chain amyloidosis in Taiwan
Source: Sci Rep. 2022 Sep 21;12:15736. doi: 10.1038/s41598-022-18990-3 (PMC9492671; doi:10.1038/s41598-022-18990-3)
Supplement: Supplementary file 1 — Supplementary Information. [file 41598_2022_18990_MOESM1_ESM.docx]

**A population-based cohort study of the epidemiology of light-chain amyloidosis in Taiwan**

Hsin-An Hou, Chao-Hsiun Tang, Choo Hua Goh, Shih-Pei Shen, Kuan-Chih Huang, Hong Qiu, Sarah Siggins, Lee Anne Rothwell, Yanfang Liu

**Supplementary Material**

**Table S1** Demographic and clinical characteristics of patients with newly diagnosed AL amyloidosis (Taiwan, 2016-2019)

|  | **Total (N=841)** | | **2016 (N=199)** | | **2017 (N=230)** | | **2018 (N=216)** | | **2019 (N=196)** | |
| --- | --- | --- | --- | --- | --- | --- | --- | --- | --- | --- |
|  | **N** | **%** | **N** | **%** | **N** | **%** | **N** | **%** | **N** | **%** |
| **Sex** |  |  |  |  |  |  |  |  |  |  |
| Male | 494 | 58.74 | 124 | 62.31 | 138 | 60 | 119 | 55.09 | 113 | 57.65 |
| Female | 347 | 41.26 | 75 | 37.69 | 92 | 40 | 97 | 44.91 | 83 | 42.35 |
| **Age, years** |  |  |  |  |  |  |  |  |  |  |
| Mean ± SD | 61.40 | 14.09 | 61.09 | 14.27 | 61.67 | 15.15 | 59.54 | 12.87 | 63.44 | 13.70 |
| <20 | * | 0.12 | 0 | 0.00 | * | 0.41 | * | 0.00 | 0 | 0.00 |
| 20-29 | 23 | 2.62 | 7 | 3.52 | 10 | 3.91 | 2 | 0.93 | 4 | 2.04 |
| 30-39 | 38 | 4.52 | 6 | 3.02 | 10 | 4.34 | 15 | 6.02 | 9 | 4.59 |
| 40-49 | 98 | 11.65 | 26 | 13.07 | 21 | 9.13 | 33 | 15.28 | 18 | 9.18 |
| 50-59 | 219 | 26.04 | 49 | 24.62 | 57 | 24.78 | 66 | 30.56 | 47 | 23.98 |
| 60-79 | 227 | 26.99 | 60 | 30.15 | 65 | 28.26 | 52 | 24.07 | 50 | 25.51 |
| 70-79 | 159 | 18.91 | 31 | 15.58 | 43 | 18.70 | 38 | 17.59 | 47 | 23.98 |
| 80+ | 77 | 9.16 | 20 | 10.05 | 24 | 10.43 | 12 | 5.56 | 21 | 10.71 |
| **CCI score** |  |  |  |  |  |  |  |  |  |  |
| Mean ± SD | 2.07 | 2.23 | 2.08 | 2.21 | 2.08 | 2.46 | 1.98 | 2.08 | 2.14 | 2.17 |
| 0 | 262 | 31.15 | 58 | 29.15 | 78 | 33.91 | 69 | 31.94 | 57 | 29.08 |
| 1 | 145 | 17.24 | 31 | 15.58 | 45 | 19.57 | 38 | 17.59 | 31 | 15.82 |
| 2 | 162 | 19.26 | 52 | 26.13 | 38 | 16.52 | 35 | 16.2 | 37 | 18.88 |
| 3 | 98 | 11.65 | 18 | 9.05 | 16 | 6.96 | 33 | 15.28 | 31 | 15.82 |
| 4+ | 174 | 20.69 | 40 | 20.1 | 53 | 23.04 | 41 | 18.98 | 40 | 20.41 |
| Any malignancy, including lymphoma and leukaemia, except malignant neoplasm of skin | 184 | 21.88 | 48 | 24.12 | 51 | 22.17 | 45 | 20.83 | 40 | 20.41 |
| Peptic Ulcer Disease | 164 | 19.5 | 44 | 22.11 | 47 | 20.43 | 37 | 17.13 | 36 | 18.37 |
| Renal Disease | 163 | 19.38 | 36 | 18.09 | 34 | 14.78 | 46 | 21.3 | 47 | 23.98 |
| Diabetes without chronic complications | 161 | 19.14 | 38 | 19.1 | 43 | 18.7 | 39 | 18.06 | 41 | 20.92 |
| Congestive Heart Failure | 112 | 13.32 | 24 | 12.06 | 30 | 13.04 | 23 | 10.65 | 35 | 17.86 |
| Cerebrovascular Disease | 88 | 10.46 | 25 | 12.56 | 27 | 11.74 | 16 | 7.41 | 20 | 10.2 |
| Chronic Pulmonary Disease | 86 | 10.23 | 17 | 8.54 | 24 | 10.43 | 23 | 10.65 | 22 | 11.22 |
| Mild Liver Disease | 82 | 9.75 | 14 | 7.04 | 25 | 10.87 | 24 | 11.11 | 19 | 9.69 |
| Diabetes with chronic complications | 77 | 9.16 | 19 | 9.55 | 18 | 7.83 | 13 | 6.02 | 27 | 13.78 |
| Dementia | 27 | 3.21 | 10 | 5.03 | 5 | 2.17 | 5 | 2.31 | 7 | 3.57 |
| Metastatic Carcinoma | 23 | 2.73 | 5 | 2.51 | 10 | 4.35 | 6 | 2.78 | ≤ 3 |  |
| Peripheral Vascular Disease | 20 | 2.38 | 7 | 3.52 | 4 | 1.74 | 5 | 2.31 | 4 | 2.04 |
| Rheumatic Disease | 18 | 2.14 | 6 | 3.02 | 5 | 2.17 | ≤ 3 |  | 4 | 2.04 |
| Paraplegia and Hemiplegia | 18 | 2.14 | 4 | 2.01 | 5 | 2.17 | 4 | 1.85 | 5 | 2.55 |
| Myocardial Infarction | 17 | 2.02 | 4 | 2.01 | 4 | 1.74 | 4 | 1.85 | 5 | 2.55 |
| Moderate or Severe Liver Disease | 4 | 0.48 | 0 | 0.00 | ≤ 3 |  | ≤ 3 |  | ≤ 3 |  |
| AIDS/HIV | ≤ 3 |  | 0 | 0.00 | 0 | 0.00 | ≤ 3 |  | 0 | 0.00 |

CCI: Charlson Comorbidity Index; SD, standard deviation,

* To protect patient privacy, all non-zero counts that were <4 were suppressed and grouped with the next category

**Table S2** Age distribution of AL Amyloidosis patients with cardiac, renal, and/or liver co-morbidities

|  | **Cardiac disease**  **N=240** | | **Liver disease**  **N=18** | | **Renal disease**  **N=195** | |
| --- | --- | --- | --- | --- | --- | --- |
| **Age group (years)** | **N** | **%** | **N** | **%** | **N** | **%** |
| <20 | 0 | 0.00 | 0 | 0.00 | 0 | 0.00 |
| 20-29 | 15* | 6.25* | 0 | 0.00 | 0 | 0.00 |
| 30-39 |  |  | 0 | 0.00 | 5 | 2.56 |
| 40-49 |  |  | 3 | 16.67 | 9 | 4.62 |
| 50-59 | 51 | 21.25 | 3 | 16.67 | 49 | 25.13 |
| 60-79 | 80 | 33.33 | 3 | 16.67 | 66 | 33.85 |
| 70-79 | 60 | 25.00 | 9 | 50.00 | 47 | 24.10 |
| 80+ | 34 | 14.17 | 0 | 0.00 | 19 | 9.74 |

*age-groups merged to protect patient privacy in groups where N<4
